# Supplementary material for: Uncovering the Number and Clonal Dynamics of Mesp1 Progenitors during Heart Morphogenesis
Source: Cell Rep. 2015 Dec 24;14(1):1–10. doi: 10.1016/j.celrep.2015.12.013 (PMC4709258; doi:10.1016/j.celrep.2015.12.013)
Supplement: Document S1. Supplemental Theory, Supplemental Experimental Procedures, and Figure S1 [file mmc1.pdf]

Cell Reports

Supplemental Information

Uncovering the Number and Clonal Dynamics of *Mesp1* Progenitors during Heart Morphogenesis

Samira Chabab, Fabienne Lescroart, Steffen Rulands, Navrita Mathiah, Benjamin D. Simons, and Cédric Blanpain

Chabab et al. Supplemental Figure S1

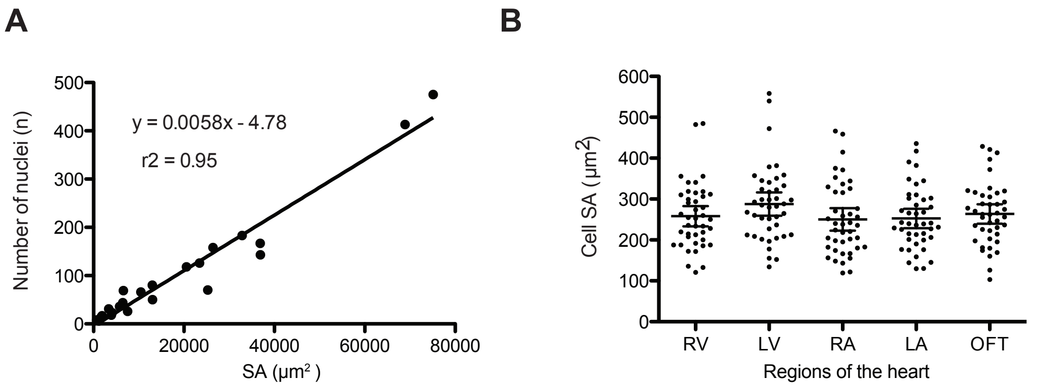

Supplemental Figure S1: Correlation between the surface area and the number of nuclei, related to Figure 1, 2 and 4

A. Correlation between the surface area (SA) (in µm^2) covered by a patch in E12.5 *Mesp1-Cre/Rosa-Confetti* hearts and the number of nuclei (n). The correlation is linear. **B**. Distribution of the measurments of the SA of CFP-expressed cells (+/- 95% CI) (in µm^2) equally distributed along the depth of the heart in the different regions of the *Mesp1-Cre/Rosa-Confetti* hearts at E12.5 showing the size of cardiac cells is constant in the different cardiac regions.

Chabab et al. Supplemental Theory: Analysis of clonal surface areas, related to Figure 1, 2 and 4

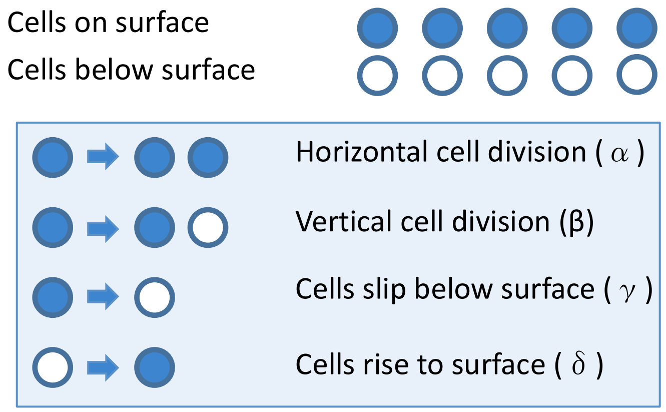

Inferring progenitor fate behaviour from surface-area data, related to Figure 1 and 4

The analysis of clonal dynamics in developing tissues typically relies on the acquisition of a statistical ensemble of clonal data. However, in three-dimensional tissues, the recovery of clonal information requires the reconstruction of serial sections, which is challenging, slow and potentially unreliable. By contrast, the distribution of clone sizes as measured by their "footprint" on the surface of tissues can be acquired at high definition from confocal microscopy of thin sections. Unfortunately, the reconstruction of total clone sizes from random sections through three-dimensional clones requires making detailed assumptions about their morphology, a canonical and unsolvable problem known in the literature as the *corpuscle problem*. Nevertheless, by focusing on cell dynamics at the surface, in this study we show that information on developmental processes can still be recovered.

To this end, we propose a simple and generic modelling framework that allows qualitative insights to be drawn from surface area data. In particular, for an equipotent population of developmental precursors, there are only a limited number of processes that change the number of cells in a labelled clone at the surface: The number of labelled progenitor cells, S, in the surface layer can increase due to tangential (in-plane) cell division, or by labelled cells

being transferred from the bulk into the surface layer (see schematic and Figure 4A). We denote the combined rate of these processes as α. Further, labelled cells may divide perpendicular to the surface so that one daughter cell stays in the surface while the other ends up below the surface (at a rate β). Finally, labelled cells may slip below the surface without division (at a rate γ), while new surface labelling might be initiated continuously as cells labelled in the bulk reach the surface at a rate δ. We can summarize this simple model in chemical notation as the following stochastic process

$$S \overset{\alpha}{\rightarrow} SS, \qquad S \overset{\beta}{\rightarrow} S\emptyset, \qquad S \overset{\gamma}{\rightarrow} \emptyset, \qquad \emptyset \overset{\delta}{\rightarrow} S$$

where $\emptyset$ denotes a "vacancy". In the parlance of population dynamics, the behaviour of labelled cell clusters in the surface layer is then effectively described by a "Galton-Watson process with immigration" {Bailey, 1964}. If the timing between consecutive events is random, and statistically uncorrelated (Markovian) with the defined average rate, and fate decisions are cell-autonomous, the chance, $P(n,t)$, of finding a labelled cell cluster with n cells is governed by a *Master equation* of the form,

$$\frac{d}{dt}P(n,t) = [\alpha\,(n-1)+\delta]P(n-1,t) + \gamma(n+1)P(n+1,t)$$
$$-[(\alpha+\gamma)n+\delta]P(n,t).$$

Solving this equation, one finds that $P(n,t)$ is defined by a negative binomial distribution,

$$P(n,t) = \binom{\delta+n-1}{n}(1-b_t)^{\delta}b_t^n,$$

where the time-dependent parameter

$$b_t = \frac{\exp[(\alpha - \gamma)t] - \alpha}{\exp\,[(\alpha - \gamma)t] - \beta}.$$

By contrast, if the timing of division of sister cells were highly synchronized, the surface area distribution would be dominated by the immigration process, leading to a distribution of the form 1/n. However, as is discussed below, in typical clonal datasets the details of cell fate decisions might be hidden in fluctuations stemming from migration processes between cell layers.

As the processes in this model do not translate to precisely defined biological processes, the inferred rates cannot easily be related to the underlying biological parameters such as the true cell division or cell migration (diffusion) rates. Rather, our approach allows the hypothesis of progenitor equipotency to be assessed, and different modes of division to be distinguished on the basis of surface clonal data alone. Note, however, that within the variability of typical clonal datasets, we cannot rule out a potential additional contribution from cell divisions leading to asymmetric fate outcome, which would simply lead to a rescaling of model parameters.

Fitting of the model, related to Figure 4

To fit the model we estimated the number of cells in a clone by dividing its surface area by the average surface area covered by cardiomyocytes (269 ± 13 (95% CI) μm^2). We then employed Maximum Likelihood Estimation as implemented in the nbinfit function of Matlab's® Statistics and Machine Learning Toolbox™. The fitted parameter values were $\delta = 4.2, 4.2, 3.8$ and $\beta_t = 0.7, 1, 0.9$ for the time points E6.25, E6.75 and E7.25, respectively.

Estimation of the number of *Mesp1* progenitors from clonal data, related to Figure 1

To estimate the number of *Mesp1* progenitors contributing to heart development we considered the portion of clone touching the surface as a random slice through the full, three-

dimensional clone. Inferring the distribution of full clone sizes from these slices is mathematically impossible, a manifestation of the *corpuscle problem*. However, assuming that the surface area of a clone is statistically representative of any other section, the average surface area fraction is an unbiased estimator of average volume fraction of a clone in the myocardium (e.g. Underwood, 1970). In other words, the average fraction of labeled *Mesp1* progenitors contributing to the surface in a clone equals the fraction of all labeled *Mesp1*+ precursors among all cardiac cells. Hence, while we cannot infer the distributions of clone sizes from surface area data, the fraction of the *average* clone size of all cardiomyocytes is equal to the average percentage of surface area, A_s, covered by a single clone. Importantly, we may therefore identify A_s with the size of a single clone. With this definition, the number of *Mesp1* progenitors contributing to the surface is then given by $N_{Mesp1} = 1/A_s$.

To calculate A_s we again make use of the 89 groups of patches, which we identified as monoclonal by statistical inference (Lescroart et al., 2014). In our previous study, hearts with three or less patches in a given color were assigned as monoclonal. However, in the assignment of hearts that are monoclonal we estimated that some 12% of these were falsely assigned as monoclonal, and likely induced in more than a single clone in a given color (Lescroart et al., 2014). To take this into account, we write the average size of visible clones in "monoclonal" hearts, A_m, as $A_m = \sum_{n=1}^{3} n\, r_n\, A_s$, where r_n denotes the fraction of hearts, which were assigned as monoclonal and were induced with *n* cells. Correspondingly, we find

$$A_s = \frac{A_m}{\sum_n n\, r_n}.$$

The frequencies r_n are given by $r_n = \sum_k L(n|k)F(k)$, with the $L(m|k)$ defining the probability of finding *n* induction events given *k* patches, and the overall frequency of hearts with *k* patches, $F(k)$, as derived in (Lescroart et al., 2014). With $A_m = 0.57 \pm 0.05$ % (all errors represent 95% CI), we obtain an average clone size of 0.5% of all CMs. Taken alone this would suggest that about 200 Mesp1+ progenitors are required to cover the total surface of a heart (1/0.005).

We next take into account the fact that some 18% of fragments do not touch the surface of the heart and have therefore not been included in the estimate of the average clone size. Consequently, if the proximity of fragments to the surface is independent of the fragment size, we underestimated the clone size by the same amount. Dividing the average clone size by 82 $\pm$ 9% (95% CI) we therefore obtain a total number of 244 +/- 26 (95% CI) (200/0.82 progenitors) *Mesp1* progenitors.

Calculation of errors in the estimation of progenitors from clonal tracing experiments, related to Figure 1

To calculate the 95% confidence interval for the number of *Mesp1* progenitors we estimated the binomial confidence interval of the proportion of surface touching cells using the Clopper-Pearson method as implemented in Matlab's binofit function (Statistical Toolbox). The relative error associated with the size of monoclonal groups of clusters was less than 2%, such that we could neglect this source of error. We obtained the final estimate for the 95% confidence intervals using the conventional formulas of error propagation (e.g. Clifford, 1973).

Estimation of the number of *Mesp1* progenitors from mosaic tracing experiments, related to Figure 2

We may also infer the number of *Mesp1* progenitors from the number of patches in mosaic labeled hearts. In this case the analysis is complicated by the fact that clones not only fragment but also merge due to a high induction frequency. In previous work, we estimated the fragmentation rate of clones to be $f = 1.6 \pm 0.2$ (95% CI) fragmentation events between labeling and analysis at E12.5, which was roughly independent of the induction time. In other words, a single induction event leads, on average, to 2.6 $\pm$ 0.2 (95% CI) patches.

Merging of clones can occur by different mechanisms: initially distant clones can become neighbors due to tissue remodeling, or cells are labeled in close proximity by chance. Given the high induction frequency we assume that the latter mechanism is by far dominant. To find the rate of labeled mergers, we made use of one of the most studied models in statistical physics. We considered an array of hexagonal cells, which are arranged on a triangular lattice (Figure 2L). Each of these cells is labeled with a probability p and remains unlabeled otherwise. In the resulting pattern, neighboring cells are often by chance labeled in a common color, such that cluster of labeled cells of varying size can emerge. The number of labeled clusters, n, is then given by the number of labeled cells divided by the average cluster size. To estimate the average size of labeled clusters we performed Monte Carlo simulations of the site percolation problem on a hexagonal lattice with 1e6 sites (Stauffer and Aharony, 1992). The average induction probability for hearts harvested at E12.5 was 0.26 ± 0.05 (95% CI), which according to the simulation, corresponds to an average initial cluster size of 2.75 ± 0.05 (95% CI) cells. With this value, the number of *Mesp1* progenitors contributing to heart development is

$$N = \frac{n\,m}{p\,(f+1)\,s},$$

where n is the number of patches in a given colour and heart, m is the average initial cluster size, f is the fragmentation rate, p is the induction probability (chimerism), and s is the fraction of surface-touching patches among all patches. Finally, we obtain the estimate that 257±24 (95% CI) *Mesp1* progenitors contribute to heart development.

Calculation of errors in the estimation of progenitors from mosaic tracing experiments, related to Figure 2

The calculations of the number of *Mesp1* progenitors and the corresponding uncertainty (95% confidence interval) were performed separately for each colour and each heart. The reported value is the average of these values. The calculations resulting in the induction frequencies

and of the average initial cluster size by Monte Carlo simulations involved large sample sizes, such that we could neglect these sources of error. Since we did not perform the Monte Carlo simulations for each colour and heart separately but for the average induction frequency in all hearts, the uncertainty in the average cluster size was estimated by the uncertainty in this average. Further, the number of patches in each colour and each heart was modelled by a Poisson distribution. The corresponding uncertainty in these estimates was obtained using Matlab's poissfit function from the Statistical Toolbox. The uncertainty of the fragmentation rate was taken from (Lescroart et al., 2014). As the different sources of error are statistically independent the uncertainties for each colour and each heart were propagated using standard formulae. Similarly, standard formulae have been used to calculate uncertainty of the final estimate from the individual values (e.g. Clifford, 1973).

Estimation of cell division rates from the clonal induction to E12.5, related to Figure 4

To determine the overall (all) and horizontal (parallel to surface) proliferation rate of *Mesp1* progenitors we respectively make use of the 8 clones in which the volume was measured and 89 clones in which the surface data was recorded, and that were identified to be monoclonal by statistical inference (Figure 4F). We begin with estimating the overall proliferation rate from the volumes of clones induced at time points E6.75 and E7.25. With the volume of a single cell at the time of analysis roughly given by $v \approx 2150\ \mu m^3$ (de Boer et al., 2012), the number of cells in a clone of volume V is given by $N = V/v$. If all progeny of the precursor labeled at time t_0 divide symmetrically, the size of clones grows exponentially, $N(t) = \exp[\lambda(t - t_0)]$, where λ is the average rate of cell division. With this, we obtain $\lambda = \ln N(t)/(t - t_0)$.

By following a similar approach we can in addition obtain the cell division rate tangential to the heart surface. For this we took the average surface area covered by a single cell to be $s = 269 \pm 13$ (95% CI) μm^2, as was measured with Fiji software (Schindelin et al., 2012) on several optical sections of mosaically labeled hearts (Figure S1B). We calculated the number

of cells within the SA covered by a clone by using the formula N = SA/s. The average SA is determined solely by cell divisions parallel to the surface. Since in a continuously expanding tissue the amount of vertical cell movements in and out of the surface layer must, on average, be equal we could neglect their contribution to the average clonal SA. With this, the "horizontal" proliferation rate of *Mesp1* expressing cells is simply given by the logarithm of the average number of surface-touching cells in a clone, divided by the time span between labeling and analysis. The "horizontal" proliferation rate is around 0.7 times per day and represents some 60-70% of all cell division (Figure 4F).

Supplemental experimental procedures

Estimation of chimerism in mosaically labeled heart

The proportion of fluorescent protein labeled cells expressed at the surface of the heart was analyzed for each channel on maximum intensity projection treated images with Zen Black software (Carl Zeiss, Inc.). The whole surface area (SA) covered by each fluorescent protein relative to the total surface of the heart was measured using Fiji software (Schindelin et al., 2012). Then, the same heart was sectioned (20-μm thick cross-sections) and the same strategy was used to determine the chimerism that occurs inside the heart.

Correlation between the number of nuclei and the surface area of a 2D-labeled cluster

The number of nuclei (counterstained with Topro-3) inside a defined cluster was counted and correlated to the SA of this cluster with Fiji software (Schindelin et al., 2012) on several optical sections of mosaically labeled hearts at E12.5 and P1.

Estimation of cardiac cells number

10 hearts from E13.5 embryos were dissected and digested individually in collagenase (3.5mg/ml) during 1.5 hour at 37°C. Enzyme activity was stopped using the final concentration of 5mM EDTA (Invitrogen). Digestion into single cells was checked microscopically and by FACS analysis where more than 80% of the cell suspension was composed by single cells. The number of cardiac cells was counted on a cell chamber counting (Neubawer, Inc) and validated with a mini automated cell counter (Moxi Z, Orflo technologies).
