# Supplementary material for: Uncovering the Number and Clonal Dynamics of Mesp1 Progenitors during Heart Morphogenesis
Source: Cell Rep. 2015 Dec 24;14(1):1–10. doi: 10.1016/j.celrep.2015.12.013 (PMC4709258; doi:10.1016/j.celrep.2015.12.013)
Supplement: Document S2. Article plus Supplemental Information [file mmc2.pdf]

Cell Reports

Report

Uncovering the Number and Clonal Dynamics of *Mesp1* Progenitors during Heart Morphogenesis

Graphical Abstract

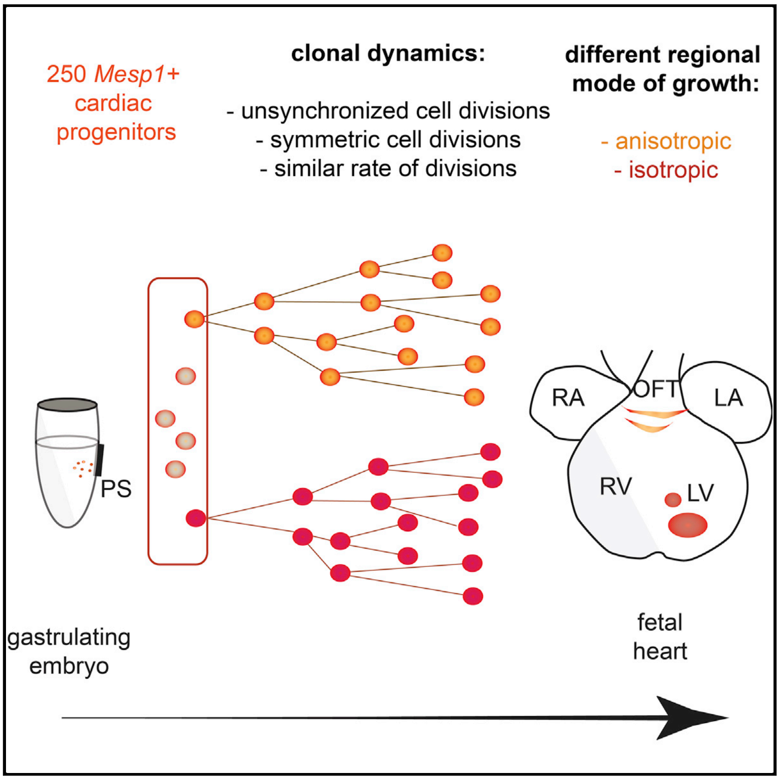

Authors

Samira Chabab, Fabienne Lescroart, Steffen Rulands, Navrita Mathiah, Benjamin D. Simons, Cédric Blanpain

Correspondence

bds10@cam.ac.uk (B.D.S.), cedric.blanpain@ulb.ac.be (C.B.)

In Brief

Chabab et al. show that about 250 *Mesp1* cardiac progenitors specified during gastrulation contribute to heart myocardium morphogenesis. Distinct subpopulations of *Mesp1* progenitors, emerging at different time points during development and leading to the formation of different regions of the heart, share similar proliferative capacities.

Highlights

- 250 *Mesp1* progenitors give rise to all cardiomyocytes
- *Mesp1* progenitors present distinct regional mode of growth
- *Mesp1* progenitors present similar clonal dynamics

CrossMark

Chabab et al., 2016, Cell Reports *14*, 1–10
January 5, 2016 ©2016 The Authors
http://dx.doi.org/10.1016/j.celrep.2015.12.013

CellPress

Cell Reports
Report

OPEN ACCESS
CellPress

Uncovering the Number and Clonal Dynamics of *Mesp1* Progenitors during Heart Morphogenesis

Samira Chabab,[1,6] Fabienne Lescroart,[1,6] Steffen Rulands,[2,3,6] Navrita Mathiah,[1] Benjamin D. Simons,[2,3,4,*] and Cédric Blanpain[1,5,*]
[1]Université Libre de Bruxelles, IRIBHM, Brussels 1070, Belgium
[2]Cavendish Laboratory, Department of Physics, University of Cambridge, J. J. Thomson Avenue, Cambridge CB3 0HE, UK
[3]The Wellcome Trust/Cancer Research UK Gurdon Institute, University of Cambridge, Tennis Court Road, Cambridge CB2 1QN, UK
[4]Wellcome Trust-Medical Research Council Stem Cell Institute, University of Cambridge, Cambridge CB2 1QR, UK
[5]WELBIO, Université Libre de Bruxelles, Brussels 1070, Belgium
[6]Co-first author
*Correspondence: bds10@cam.ac.uk (B.D.S.), cedric.blanpain@ulb.ac.be (C.B.)
http://dx.doi.org/10.1016/j.celrep.2015.12.013
This is an open access article under the CC BY license (http://creativecommons.org/licenses/by/4.0/).

SUMMARY

The heart arises from distinct sources of cardiac progenitors that independently express *Mesp1* during gastrulation. The precise number of *Mesp1* progenitors that are specified during the early stage of gastrulation, and their clonal behavior during heart morphogenesis, is currently unknown. Here, we used clonal and mosaic tracing of *Mesp1*-expressing cells combined with quantitative biophysical analysis of the clonal data to define the number of cardiac progenitors and their mode of growth during heart development. Our data indicate that the myocardial layer of the heart derive from ∼250 *Mesp1*-expressing cardiac progenitors born during gastrulation. Despite arising at different time points and contributing to different heart regions, the temporally distinct cardiac progenitors present very similar clonal dynamics. These results provide insights into the number of cardiac progenitors and their mode of growth and open up avenues to decipher the clonal dynamics of progenitors in other organs and tissues.

INTRODUCTION

The three germ layers, which give rise to all future tissues and organs of the embryo, are generated during gastrulation (Tam and Beddington, 1992). To ensure the harmonious morphogenesis of the different organs, it is crucial that a precise number of progenitors for each organ and tissue is specified at this critical stage of development. Moreover, once those progenitors are specified, they must migrate and proliferate to expand the pool of progenitors that will eventually differentiate into the different cell types that make up the different organs and tissues.

The heart represents one of the first functional organs to form during development (Garry and Olson, 2006). Retrospective clonal analysis, in which cardiac progenitors are labeled randomly during cardiac development, has suggested that heart morphogenesis involves two distinct groups of cardiac progenitors called first and second heart field (FHF and SHF, respectively) (Buckingham et al., 2005). FHF cardiac progenitors contribute to the formation of the left ventricle (LV) whereas SHF progenitors give rise to the outflow and inflow tracts (OFT and IFT, respectively). The other cardiac regions, the right ventricle (RV) and the left and right atria (LA and RA, respectively), arise from both heart fields (Buckingham et al., 2005; Meilhac et al., 2004a). Heart development begins during the initial stage of gastrulation, when cells within the primitive streak (PS) begin to express *Mesp1*, undergo epithelial-to-mesenchymal transition (EMT), and migrate toward the anterolateral part of the embryo, where they form the cardiac crescent (Buckingham et al., 2005). *Mesp1-Cre* lineage tracing has shown that the majority of cardiac cells, including FHF and SHF derivatives, originate from *Mesp1*-expressing cells (Lescroart et al., 2014; Saga et al., 1999). However, clonal analysis of *Mesp1*-expressing cells demonstrates that two temporally distinct pools of *Mesp1* progenitors sequentially give rise to the FHF and then the SHF progenitors (Lescroart et al., 2014). It remains unclear how many FHF and SHF progenitors are generated during gastrulation; what their respective mode of growth is, as defined by the shape and orientation of the clones reflecting isotropic versus anisotropic growth; and how they balance proliferation and differentiation (clonal dynamics), from their departure from the PS during gastrulation to development of an adult heart.

Here, using a multidisciplinary approach involving *Mesp1*-specific multicolor mosaic and clonal lineage tracing to mark single *Mesp1*-expressing cells combined with biophysical analysis of their fate during development and postnatal life, we defined the number of *Mesp1* cardiac progenitors that are specified during the early stage of gastrulation and their individual contribution to the morphogenesis of the heart. We found that ∼250 *Mesp1* progenitors contribute to cardiac morphogenesis. Surprisingly, we found that, despite their emergence at different time points during gastrulation, their early commitment to distinct heart regions, and their contrasting morphological growth characteristics, the proliferative capacity of the different *Mesp1* progenitors is remarkably similar.

RESULTS

Two Hundred Fifty *Mesp1* Progenitors Contribute to Myocardial Morphogenesis

To infer the number of *Mesp1* cardiovascular progenitors that contribute to the formation of the myocardial layer of the different heart regions during mouse embryonic development, which is composed of cardiomyocytes (CMs), we performed clonal analysis of single *Mesp1*-expressing (*Mesp1*+) cells and determined their individual contribution to heart morphogenesis (Lescroart et al., 2014). A low dose of doxycycline was administrated to *Mesp1-rtTA/TetO-Cre/Rosa-confetti* pregnant females between embryonic day (E)6.25 and E7.25, and embryos were analyzed at E12.5 for the contribution of single *Mesp1*+ cells (n = 89 clones) (Figures 1A and 1B) (Lescroart et al., 2014). Clonal analysis is complicated by the fact that the progeny of single *Mesp1* progenitors can "fragment" into disconnected clusters of lineage-labeled cells as heart morphogenesis proceeds. However, we have previously showed that stochastic modeling of the induction and clone fragmentation processes can be used to reliably infer the fragmentation rate of cells arising from *Mesp1* progenitors during cardiac development (2.6 ± 0.2 fragments per clone) (Lescroart et al., 2014). With this information in hand, treating each confetti color as independent, we were able to use statistical inference to separate labeled hearts into those involving a single clonal induction event from those involving the multiple progenitors. In particular, we found that hearts with fewer than four fragments in a given color of the confetti reporter transgene are, with known confidence (88%), monoclonal. With this, we then filtered for monoclonal hearts, leaving us with a set of well-defined clonal lineages (Lescroart et al., 2014).

The heart is a complex organ with a three-dimensional (3D) organization. The recovery of clonal information in 3D requires reconstruction of serial sections or 3D imaging, which are still challenging and time consuming. We can obtain information on the clonal contribution of cardiac progenitors by using their "clonal footprint" on the surface of the heart, which can then be acquired at high definition from confocal microscopy. However, reconstruction of the clone in three dimensions from their "footprint" requires making assumptions about their morphology or depth, a canonical problem known as the corpuscle problem (Wicksell, 1925). Since the total surface area (SA) of individual clones was found to be linearly correlated with the number of constituent cells on the surface of the heart (Figure S1), the SA could be taken as a proxy of the individual area clone size.

Although we did not know the depth of clones, we could make use of the fact that the average volume fraction of clones is well estimated by the average SA fraction of the heart surface (Supplemental Theory) (Underwood, 1970). Thus, to estimate the number of *Mesp1*+ cells that contribute to heart morphogenesis, we assessed the size distribution of SAs occupied by individual clones in monoclonal hearts induced at E6.25, E6.75, or E7.25 (Figures 1C–1F). The analysis of the SA is mainly informative about the myocardial layer that represents the bulk of the cardiac wall composed by CMs. Then, if we assume that the proliferative capacity of individual *Mesp1*-expressing cells is similar (discussed later), from the average surface fraction of the heart covered by a single clone, A_{single}, the number of *Mesp1* progenitors that contribute to the surface is simply given by $1/A_{single}$. To calculate A_{single}, we measured the fragments' SAs for all induction times (E6.25, E6.75, and E7.25), which were then normalized to the total SA of the heart (Figures 1C–1F). By summing up the SAs of monoclonal fragments, we found that, on average, each *Mesp1* progenitor contributes to 0.57% ± 0.05% (95% confidence interval [CI]) of the total SA of the heart (Figures 1C–1F).

To determine how much the external SA is representative of the total number of CMs, we assessed the relative contribution of *Mesp1* progenitors to the myocardial cells located inside the heart (Figures 1G and 1H). 3D reconstruction of confocal analyses of *Mesp1-rtTA/TetO-Cre/Rosa-confetti* hearts at E12.5 showed that the majority of fragments (82 ± 9%; 95% CI) located inside the heart made contact with the surface of the heart (Figures 1G and 1H). Correcting for the minority of fragments that remain below the surface of the heart, and taking into account the distribution of fragment numbers in individual clones (see Experimental Procedures), we estimated that ~244 ± 26 (95% CI) *Mesp1* progenitors contribute to heart development (Figure 1I).

Insights from Multicolor Mosaic *Mesp1* Lineage Tracing

To independently validate the number of *Mesp1* progenitors inferred from the study of monoclonal hearts and gain further insight into the growth of the tissue, we quantified the size and morphology of fluorescently labeled surface patches that were obtained in *Mesp1-Cre/Rosa-Confetti* hearts at E12.5 when labeled at higher induction frequency (Figures 2A and 2B). The very transient expression of the *Mesp1-Cre* (about 24 hr) during embryonic development (Saga et al., 1999) did not induce color conversion of the Rosa-confetti reporter system, which can occur upon Cre re-expression in adult tissue and would be visible as GFP clones within a yellow fluorescent protein (YFP) clone or as CFP clones within a red fluorescent protein (RFP) clone (Schepers et al., 2012). Such clones were not observed in *Mesp1-Cre/Rosa-confetti*-labeled hearts (Figures 2C–2F). However, at this level of mosaicism, one cell cluster labeled in a given color can, in principle, be derived from the fusion of two or more independent progenitors induced with the same color. As with fragmentation, the rate of such clone merger or fusion events can be resolved by making use of statistical methods.

To begin, we quantified the recombination frequency of three of the confetti reporter constructs (CFP, YFP, and RFP), both at the surface and inside the mosaic heart, at E12.5 (Figures 2C–2K). Patches bearing nuclear GFP labeling were discarded, as their SA is much more complicated to quantify due to the nuclear nature of labeling. We also quantified the degree of chimerism in fluorescent labeling by assessing the ratio of color-labeled cardiac cells to unlabeled cells in mosaic hearts. Mosaic induction at high frequency showed that the proportions of CFP, YFP, and RFP were similar, leading to more than 50% of the cardiac surface becoming fluorescently labeled (Figures 2C–2K). In addition, the percentage of fluorescent labeling was similar at the surface and inside the heart at E12.5 (Figures 2G and 2H), indicating that the percentage of chimerism calculated at the surface of the heart is representative of the heart as a whole (Figures 2C–2K).

OPEN
ACCESS
CellPress

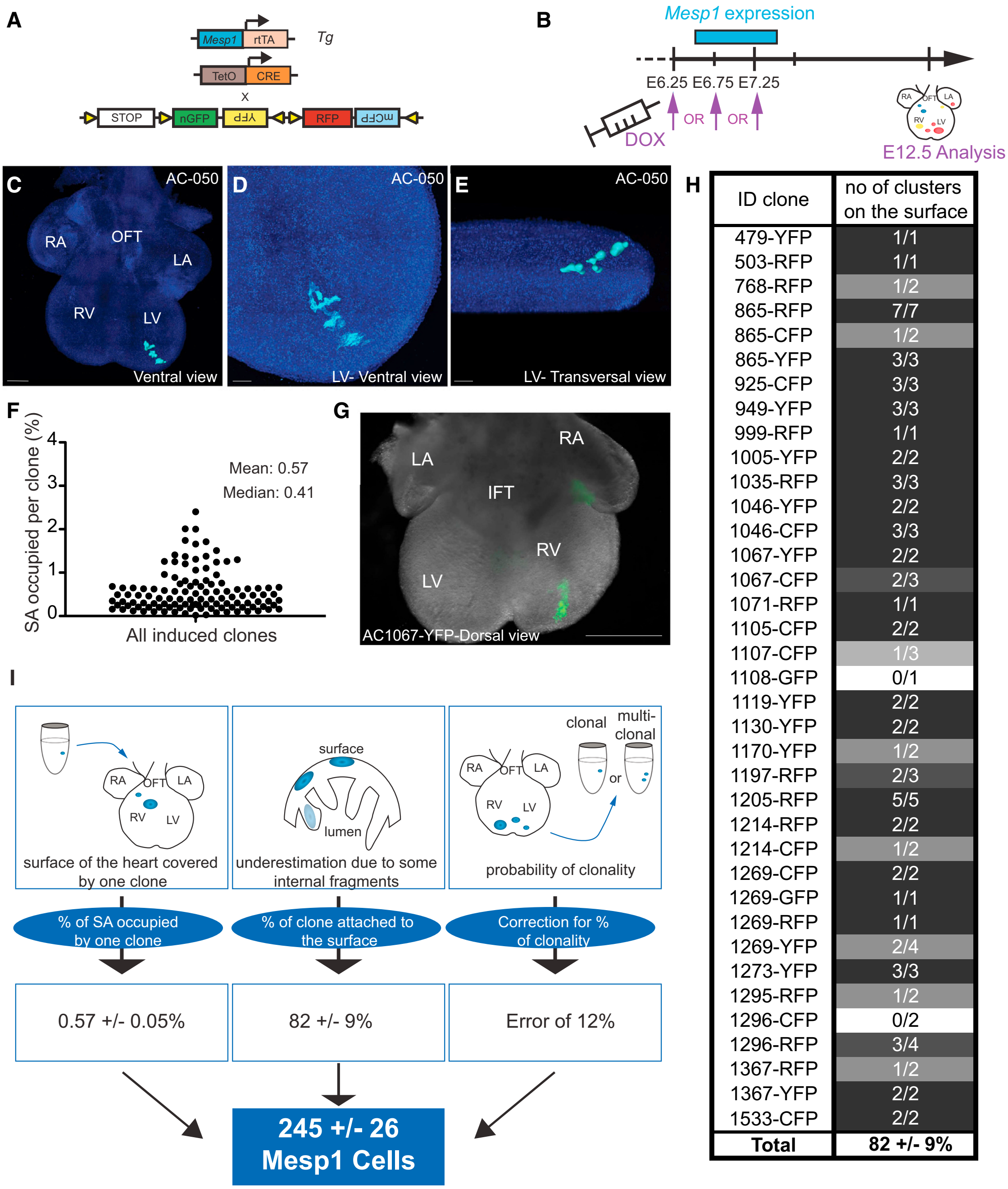

ID clone	no of clusters on the surface
479-YFP	1/1
503-RFP	1/1
768-RFP	1/2
865-RFP	7/7
865-CFP	1/2
865-YFP	3/3
925-CFP	3/3
949-YFP	3/3
999-RFP	1/1
1005-YFP	2/2
1035-RFP	3/3
1046-YFP	2/2
1046-CFP	3/3
1067-YFP	2/2
1067-CFP	2/3
1071-RFP	1/1
1105-CFP	2/2
1107-CFP	1/3
1108-GFP	0/1
1119-YFP	2/2
1130-YFP	2/2
1170-YFP	1/2
1197-RFP	2/3
1205-RFP	5/5
1214-RFP	2/2
1214-CFP	1/2
1269-CFP	2/2
1269-GFP	1/1
1269-RFP	1/1
1269-YFP	2/4
1273-YFP	3/3
1295-RFP	1/2
1296-CFP	0/2
1296-RFP	3/4
1367-RFP	1/2
1367-YFP	2/2
1533-CFP	2/2
**Total**	**82 +/- 9%**

Figure 1. Two Hundred Fifty *Mesp1* Progenitors Contribute to Cardiac Morphogenesis

(A) Scheme of the strategy used for the clonal tracing of *Mesp1*+ progenitors to assess their contribution and their mode of growth through development.

(B) A low dose of doxycycline (DOX) was injected into pregnant females at different time points of development (E6.25, E6.75, or E7.25; the word "or" is shown in all capital letters), and the hearts were analyzed at E12.5.

(C–E) Example of an E12.5 heart, induced at E6.25 (C; high magnification in D; transversal view in E) showing the shape and the fragmentation of a single FHF-derived *Mesp1* progenitor. The number on the upper right in each panel refers to the ID of the labeled heart. Scale bars, 300 μm in (C) and 100 μm in (D) and (E).

(F) Distribution of the SAs of each clone (n = 89) relative to the total surface of the heart (E6.25, E6.75, and E7.25).

(G) Example of an induced heart that showed contribution of a single *Mesp1*+ cell through all the depth of the heart. Scale bar, 500 μm.

(H) Table showing, for each induced and sectioned heart, the fraction of clones that are found at the surface of the heart.

(I) Method used to calculate the number of *Mesp1*+ cells required for heart formation. The initial number of cardiac progenitors (245 ± 26) was defined by the average SA covered by one clone, corrected with the percentage of clones that did not present labeled area at the surface of the heart and with the error that we made by including polyclonal labeled hearts to the analysis. Errors indicate means ± 95% CI; n = 89.

See Supplemental Theory and Figure S1.

OPEN ACCESS
CellPress

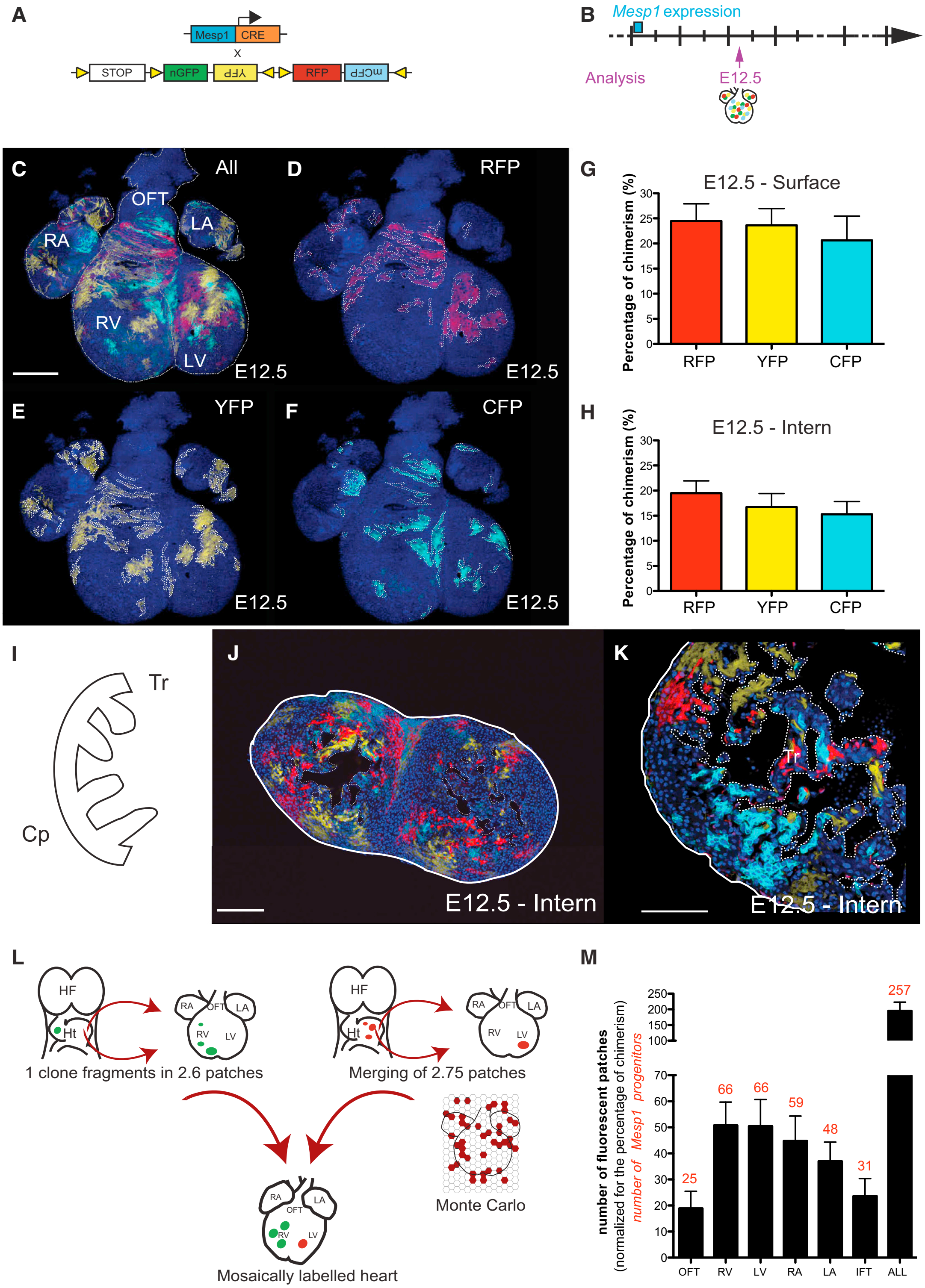

Figure 2. Insights from Multicolor Mosaic *Mesp1* Lineage Tracing

(A) Scheme of the strategy used for mosaic labeling of *Mesp1*+ cells with different fluorescent proteins.

(B) Hearts were analyzed at E12.5 (n = 4) to assess the behavior of several *Mesp1*+ cells on mosaically labeled heart.

(C–F) Picture of the surface of a *Mesp1-Cre/Rosa-Confetti* heart at E12.5 is shown at low magnification (C). The pictures of each independent channel (RFP in D, YFP in E, and CFP in F) show the different clusters, underlined by white dotted lines.

(legend continued on next page)

Trabeculae are myocardial protrusions that develop in the lumen of the ventricles and are thought to improve myocardium oxygenation (Wessels and Sedmera, 2003). Our analysis revealed that, at E12.5, trabeculum contained cells that expressed at least three of the four fluorescent proteins (Figures 2I–2K), demonstrating the polyclonal origin of the mouse heart trabeculum, a possibly conserved feature across vertebrates (Gupta and Poss, 2012).

Determining the number of *Mesp1* progenitors from the number of clusters in mosaic hearts is complicated by fragmentation and merging of cell clusters (Figure 2L). Previously, we have shown that a single *Mesp1* progenitor gives rise, on average, to 2.6 ± 0.2 (95% CI) distinct (i.e., separated) clusters (Lescroart et al., 2014). At the same time, at higher labeling density, independently labeled clusters of the same color can merge during heart morphogenesis. Similarly, cells of identical color are frequently induced next to each other. Indeed, given the high induction frequency, we assumed that this process of neighbor co-labeling is much more prominent than the fusion of clones during later development. Therefore, to estimate the rate of clone merger events in the background of clone fragmentation, we performed Monte Carlo simulations of the random labeling of an "idealized two-dimensional tissue" representing the field of *Mesp1* progenitors (Figure 2L). With cells on a triangular lattice labeled with a probability corresponding to the same percentage of chimerism observed in the *Mesp1-Cre/Rosa-Confetti* experiment, we computed the size distribution of clusters of cells labeled in the same color (Figure 2L). From these simulations, we inferred that labeled cells fuse, on average, to compounds of ~2.75 ± 0.05 (95% CI) at E12.5 (Figure 2L; Supplemental Theory). Furthermore, from this numerical simulation, we found that the fragmentation rate is approximately equal to the merging rate, suggesting that the total number of clusters provides a good estimate of the actual number of *Mesp1* progenitors.

With the number of clusters that covered the heart surface and the rate of fragmentation and merging defined, we could then estimate the number of *Mesp1+* progenitors that contribute to the heart morphogenesis. Confocal analysis of hearts at E12.5 showed that hearts were covered by 195 ± 13 (95% CI) clusters on average (Figure 2M), which, upon normalization for the percentage of chimerism and the fraction of inner fragments that were not visible from the outside (18% ± 9%; 95% CI) (Figure 1H), led to an estimate of 257 ± 24 (95% CI) *Mesp1* progenitors that contribute to the morphogenesis of the myocardial layer of the heart (Figure 2M). This number of *Mesp1* progenitors inferred from mosaic analysis is in remarkably good agreement with the number obtained from the analysis of monoclonal clusters. Our analysis also suggests that the number of *Mesp1* progenitors committed to the different cardiac chambers scales in proportion to the chamber size in the mature heart (Figure 2M).

Mesp1 Progenitors Present a Different Regional Mode of Growth

Using retrospective clonal analysis, it has been shown that cardiac progenitors generate clones of highly variable shapes at E12.5 (Meilhac et al., 2004b). However, it remains unclear whether the different morphology of clones depends on their FHF or SHF origin, on the timing of their specification, or on the time points during development at which these clones have been analyzed. Interestingly, we showed that the shape of clusters varies depending on their regional location and with the time point of analysis (E12.5, n = 4; postnatal day [P]1, n = 3; or adult stage, n = 3) (Figures 3A–3I). At E12.5, clones in the OFT are highly anisotropic and oriented along the circumference of the cavities, as previously reported (Meilhac et al., 2004b) (Figures 3C and 3D). In the ventricles, labeled cells cluster in patches of different shapes, with some (particularly in the LV) showing a rectangular shape in which the long side was oriented toward the apex, consistent with isotropic growth (Figures 3C and 3E). Moreover, *Mesp1*-derived clones in the RV were more heterogeneous in shape, with different orientations depending on the ventricle regions (Figures 3C and 3F). In particular, clones of the upper right RV presented a similar orientation to that found in the OFT, suggesting that this RV region experienced a mode of growth that was different from that of the rest of the ventricles (Figures 3C and 3F). Finally, the atria showed clusters of labeled cells that also followed the overall orientation of the cavity (Figures 3C and 3G).

In contrast, analysis of clones at P1 showed that, while the general morphology was conserved, their shapes (in particular, in the RV) were different from those observed at E12.5. Clones in the ventricles at P1 were even further elongated and enlarged (Figure 3H). Finally, analysis in adult mice showed that the shape of the clones did not change significantly from P1 (Figure 3I). These data indicate that the spatial mode of growth of *Mesp1*-derived progenitors differs significantly between heart regions and the time points in which they are analyzed, suggesting that heart remodeling that occurs during cardiogenesis influences the regional mode of growth.

(G and H) Proportion of labeled cells expressing one of the fluorescent proteins (RFP, YFP, or CFP) on the surface of the heart at E12.5 (n = 9) (G) and on sections (n = 23) (H), showing that the chimerism measured at the surface is representative of the whole heart.

(I) Scheme of the ventricular wall at E12.5. The myocardium is composed of a compact layer (Cp) and trabeculae (Tr).

(J and K) Sections of *Mesp1-Cre/Rosa-Confetti* heart at E12.5 showing that the trabeculae (Tr) contain cells labeled with more than one fluorescent protein, demonstrating their polyclonal origin.

(L) Illustration indicating that the fragmentation rate and the merging rate contribute to the final number of fluorescently labeled patches. Here, these two numbers are almost equivalent. The merging rate was calculated by computer simulation (Monte Carlo) of randomly labeling virtual cells. Labeled cells are marked red, and unlabeled cells are marked white. The size distribution of labeled clusters of cells determines the degree of merging. HF, head fold; Ht, heart tube.

(M) Mean number of labeled *Mesp1* patches per heart regions or in the whole heart normalized to the percentage of chimerism (n = 587). Corrected values (for the percentage of cells that do not colonize the surface of the heart) are indicated in red and show that the heart is formed by around 250 *Mesp1* progenitors. ALL, total number of patches/progenitors across all regions.

Error bars indicate means ± 95% CI. Scale bars, 500 μm in (C)–(F); 200 μm in (J); and 150 μm in (K). See also Supplemental Theory.

OPEN
ACCESS
CellPress

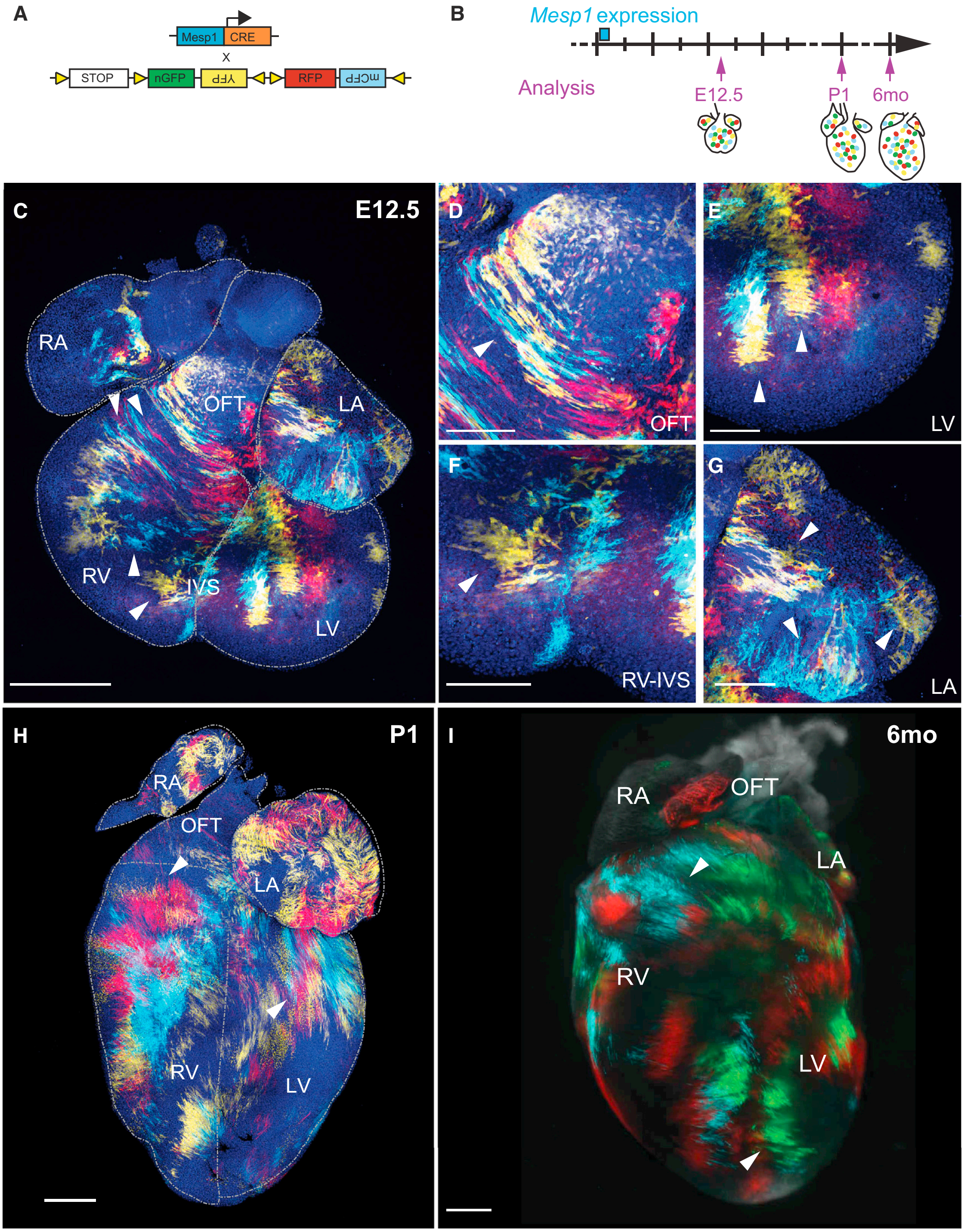

Figure 3. *Mesp1* Progenitors Present Different Regional Modes of Growth

(A) Scheme of the strategy used for mosaic labeling of *Mesp1*+ progenitors with different fluorescent proteins.

(B) Hearts were collected from E12.5 to the adult stage.

(C–G) Confocal images of a mosaic-labeled *Mesp1-Cre/Rosa-Confetti* heart at E12.5. (C) Low magnification showing the entire heart. (D–G) Higher magnification of the OFT (D), LV (E), RV-IVS (inter-ventricular septum) (F), and LA (G), showing the different shapes of *Mesp1*-derived patches according to their regional localization.

(H and I) Confocal images of *Mesp1-Cre/Rosa-Confetti* heart after birth (P1) (H) and macroscopic picture of a mosaically labeled heart at 6 months old (6mo) (I), showing that the shape of the clones was conserved during the maturation of heart.

Arrowheads indicate the shape of interesting clones. Scale bars, 500 μm. For E12.5, n = 4; for P1, n = 3; for 6 months old, n = 3.

OPEN
ACCESS
CellPress

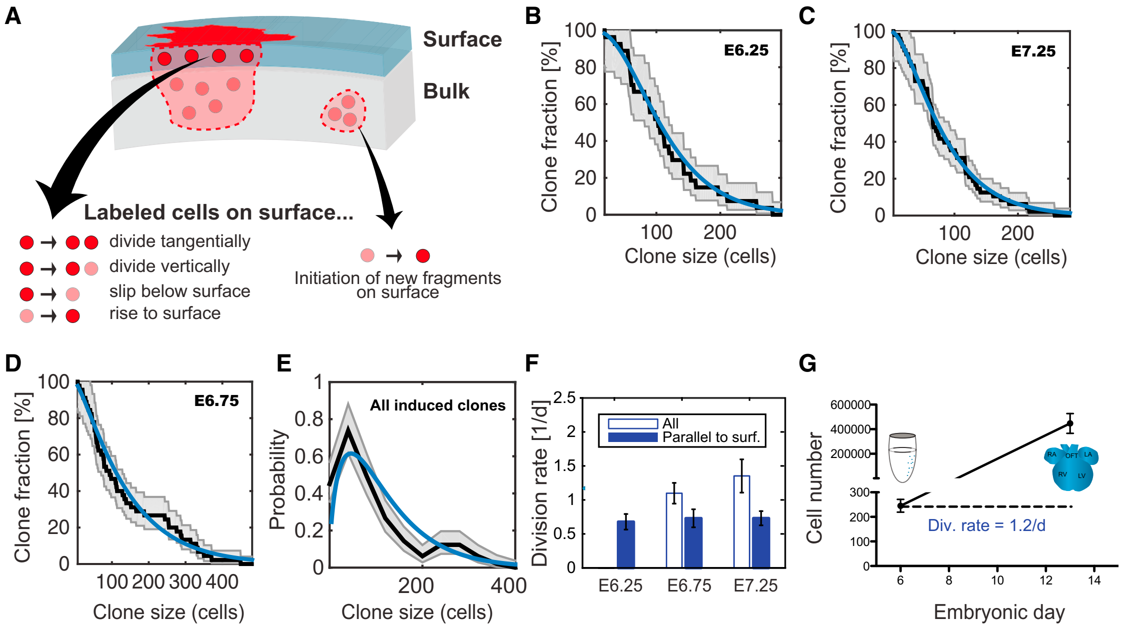

Figure 4. Different *Mesp1* Progenitors Show Similar Proliferative Potential

(A) Illustration showing a modeling framework allowing to extract qualitative biological information from SA data by taking into account vertical movements of cells and initiation of new surface-touching fragments.

(B and C) Cumulative clone size distribution (black line) and one-sigma CIs (gray line) of E6.25-induced (FHF-enriched population) (B) and E7.25-induced (SHF-enriched population) (C) monoclonal-labeled hearts showing that these 2 populations of cardiac progenitors present similar proliferation potential. The blue line corresponds to the negative binomial distribution of the theoretical model of equipotent cells dividing symmetrically.

(D) Cumulative clone size distribution (black line) and one-sigma CIs (gray line) of E6.75-induced (FHF-enriched population) monoclonal-labeled hearts, showing that cells do not follow the distribution of the theoretical model (blue line) and suggesting the presence of a highly proliferative subpopulation of cardiac progenitors at this time point.

(E) Overall probability density of clone sizes (black line) with one-sigma CIs (gray line) showing a bimodality in the distribution and confirming the presence of progenitors that have similar potential of proliferation as well as a highly proliferative subpopulation. The blue line corresponds to the theoretical model.

(F) Division rate in the population of monoclonally induced cells at E6.25 (FHF-enriched population), E6.75 (FHF-enriched population), and E7.25 (SHF-enriched population). Blue column is for overall division rate, while white column is for division rate parallel to the surface layer (surf.) of the heart at E12.5. Error bars indicate means ± SEM.

(G) Graph depicting the increase of cardiac cell numbers from E6.5 to E13.5. Error bars indicate means ± 95% CI; n = 10. Div,. division.

See also Supplemental Theory and Figure S1.

Different *Mesp1* Progenitors Show Similar Clonal Dynamics

Although the variable patterns of clonal expansion reflect particular morphological characteristics of the different heart regions, the question of whether the proliferative capacity of *Mesp1* progenitors also correlates with position remains open. To define the clonal dynamics of cardiac progenitors, we assessed the size of individual clones derived from *Mesp1*+ progenitors induced at different times during gastrulation (E6.25, E6.75, and E7.25).

To infer information on the fate behavior of cardiac precursors, we made use of the resulting distribution of the SAs of clones (n = 89) (Figure 1). As emphasized earlier, the random nature of the intersection of clones with the heart surface makes recovery of the corresponding total (3D) clone size mathematically infeasible. However, by focusing on cell dynamics at the heart surface alone, information on cell fate behavior can still be recovered. In particular, we can recover the clonal dynamics, i.e., the rate of cell proliferation and whether cell division leads to two progenitors or one progenitor and one differentiated non-proliferative cell (see Supplemental Theory). More specifically, to model the dynamics of individual clones at the surface of the heart, we note that only a limited number of processes can occur (Figure 4A; Supplemental Theory): upon division, cells in the surface layer can undergo horizontal cell division or vertical cell division; a labeled cell can rise to the surface or can slip below the surface. This means that the clone surface increases and decreases in size at rates proportional to the size of the labeled SA. Furthermore, since *Mesp1*+ progenitors labeled during gastrulation are not necessarily located on the heart surface during the early stage of cardiac morphogenesis, the labeled cells at the surface may arise at any time after their labeling.

The combination of these processes is known as a Galton-Watson process with immigration. Its solution is known to be the negative binomial distribution (Bailey, 1964). Interestingly, this model (Figure 4A) describes well the observed SAs of clones induced at E6.25 (Figure 4B) and E7.25 (Figure 4C) and suggests that the seemingly broad distribution of SAs can be explained by the stochastic nature of cell divisions and migration of cells to and from the surface layer. Indeed, when taking into account

the different time spans between labeling and analysis, progenitors defined at each of the two time points behave in a statistically similar manner. Although a small contribution from asymmetric divisions at this stage of development cannot be ruled out, the coincidence of the measured size distribution with the theoretical prediction suggests that clonal dynamics are dominated by symmetric (proliferative) cell divisions and that division timing of sister cells is not highly synchronized (see Supplemental Theory). Indeed, this modeling scheme provides a general framework to study SA data in developing tissues. While it does not allow us to decipher quantitatively the relative contribution of cell migration processes and division rates, it allows the fundamental rules of proliferation (equipotency, symmetric amplification) in these tissues to be inferred.

Curiously, for cells induced at E6.75, we found that the distribution of clonal SAs deviates from the model prediction for large SAs (Figure 4D). Indeed, the overall distribution of clonal SAs exhibits bimodality, suggesting the existence of a more proliferative subpopulation of cardiac progenitors induced at E6.75, which is not specific to any region within the heart (Figure 4E). However, even at this time point, the distribution of small clones is, again, remarkably similar to that of the other time points.

To estimate the cell division rate of *Mesp1*+ progenitors, we analyzed the volume covered by eight clones induced at E6.75 or E7.25. First, we calculated the total number of cells contained in a given clonal volume by dividing the sum of the volumes of monoclonal fragments by the average volume occupied by one cardiac cell (2,150 μm^3) (de Boer et al., 2012). Then, assuming that development is dominated by symmetric cell divisions, the division rate is given by the logarithm of the average number of cells in a clone divided by the time span between induction and analysis (see Supplemental Theory). Using this approach, we found that *Mesp1* progenitors divide, on average, between 1,1 ± 0,15 times per day from E6.75 to E12.5 and 1,4 ± 0,25 per day from E7.25 to E12.5 (Figure 4F). The difference in the proliferation rate between these two populations of *Mesp1*+ cells is not significantly different suggesting that temporally distinct *Mesp1* progenitors present very similar proliferation potential from the time of their specification to E12.5.

Similarly, we obtained the effective rate of "horizontal" divisions in the surface layer (Figure 4F) of the heart from the SA covered by each of the 89 clones. The average SA is determined solely by cell divisions parallel to the surface. Since, in a continuously expanding tissue, the amount of vertical cell movements in and out of the surface layer must, on average, be equal we could neglect their contribution to the average clonal SA. With this in mind, the "horizontal" proliferation rate of *Mesp1* expressing cells is simply given by the logarithm of the average number of surface-touching cells in a clone, divided by the time span between labeling and analysis. The "horizontal" proliferation rate is then estimated at around 0.7 per day and represents some 60%–70% of all cell divisions (Figure 4F).

Finally, to independently confirm the overall proliferation rate, we counted the number of cells at E13.5 after single-cell dissociation of fetal hearts and found that the heart is composed of 447000 ± 80000 (95% CI) cardiac cells (n = 10) at this time point (Figure 4G). With the number of *Mesp1* progenitors obtained from the lineage-tracing experiments, we deduced that *Mesp1*+ cells divide, on average, 1.2 times per day from E6.75 to E13.5, in very good agreement with the proliferation rate inferred from the volume of clones induced at E6.25 and E7.25 (Figures 4F and 4G). Interestingly, our data showed that, despite the distinct temporal origin of FHF and SHF progenitors, their different regional contributions, and the difference in their clone shape, the different *Mesp1* progenitors present very similar clonal dynamics. This suggests that the variability in surface clone size can be attributed to the stochastic nature of cell divisions and migration of cells to and from the surface layer.

DISCUSSION

By combining mosaic tracing and clonal analysis, we have resolved the number of *Mesp1* progenitors and their individual clonal dynamics during cardiac development. We found that ~250 *Mesp1* progenitors specified during gastrulation contribute to the development of the myocardial cells of the heart. Furthermore, despite arising at different times in development and contributing to different regions, the temporally distinct cardiac progenitors present very similar clonal dynamics.

While retrospective clonal analysis has suggested that the heart is formed by 140 progenitors (Meilhac et al., 2004b), our prospective clonal analysis and mosaic tracing indicate, instead, that heart development is rather mediated by 250 *Mesp1* progenitors. As retrospective clonal analysis is based on spontaneous mutations of a reporter gene that occur randomly from fertilization to the time point at which the mice are analyzed, it is difficult to date with precision when the founding mutation occurs. Clone dating is usually inferred from the sum of the size of all clusters of marked cells derived from a clonal event. The inference between birth dating and total clone size assumes that all progenitors divide synchronously at the same rate at different locations of the future heart.

Our prospective clonal analysis based on the temporally regulated *Mesp1* tracing experiments suggest that several of the previous assumptions made to estimate the number of cardiac progenitors by retrospective clonal analysis should be revised. We, and others, found no evidence of a common progenitor for the FHF and SHF but, instead, found that different pools of progenitors contribute to the morphogenesis of the different cardiac regions (Devine et al., 2014; Lescroart et al., 2014). These data suggest that the mutation in the few clones that were common from the different heart fields must have occurred before *Mesp1* expression. In addition, we found that the apparent heterogeneity of surface clone size at E12.5 is not derived from the differential proliferative capacity of *Mesp1* progenitors but can be fully explained by the stochastic transfer of cells in and out of the surface layer. Indeed, our analysis suggests that, over the developmental time course, different *Mesp1*-expressing cells share a surprising similar proliferative potential, suggesting that progenitors may follow a largely similar cell-intrinsic program. However, the homogeneity in proliferative potential does not preclude the possibility that cells progress at different rates through this program, as indicated by recent bromodeoxyuridine (BrdU) analyses, which reveal spatial heterogeneity in local proliferative activity at different times in development (de Boer et al., 2012).

The mosaically labeled hearts also reveal modes of growth that are specific to the different regions of the heart. The origin of the anisotropic growth in the OFT or isotropic growth in the other regions is still poorly understood. Such anisotropy can be driven by polarized cell divisions and/or by mechanical forces. While absence of blood flow does not impair the general morphology of the heart in vertebrates, it is possible that cardiac contraction and blood flow can contribute to the anisotropic shape of the clones, as they do during valve and trabeculae formation or ventricle remodeling (Auman et al., 2007; Koushik et al., 2001). Future studies will be required to elucidate this important question.

In contrast to clonal analysis, multicolor mosaic tracing of the heart provides an opportunity to gather information on the regional contribution of each specific cardiac progenitor and allows the visualization of the contribution of around one half of the cardiac progenitors at once in a single heart. The number of *Mesp1* progenitors is not conserved across the animal kingdom, as in ascidian *C. Intestinalis*, the heart derives from only two *Mesp*+ progenitors (Satou et al., 2004), suggesting that the number of progenitors increase with the size and complexity of the heart.

The final size of the heart may intimately depend on the number of cardiovascular progenitors that are initially specified. However, little is known about the signaling that dictates the number of cardiac progenitors or the balance between their proliferation and terminal differentiation during heart morphogenesis. Future lineage ablation studies will be required to assess the importance of specifying the correct number of progenitors at the correct time, as well as the potential plasticity of the regionally distinct *Mesp1* progenitors during heart morphogenesis.

In conclusion, we have developed an experimental and theoretical framework to define the number of cardiac progenitors, their spatial pattern of growth, and proliferation dynamics during heart morphogenesis. The statistical approach is general and can be adapted to define the number and proliferation dynamics of progenitors in other organs and tissues, such as the liver, pancreas, or different brain regions.

EXPERIMENTAL PROCEDURES

Mice

Mesp1-Cre (Saga et al., 1999) mice were obtained from Margaret Buckingham. *Rosa-Confetti* mice were kindly provided by Hans Clevers (Snippert et al., 2010). *TetO-Cre* mice (Perl et al., 2002) were provided by Andras Nagy. *Mesp1-rtTA* transgenic mice were previously described (Lescroart et al., 2014). Mice colonies were maintained in a certified animal facility in accordance with European guidelines. These experiments were approved by the local ethical committee under the protocol number LA1230332(CEBEA).

Clonal Analysis

Mesp1-rtTA/TetO-Cre/Rosa-Confetti-induced hearts that we analyzed were produced as previously described (Lescroart et al., 2014).

Clonal SA Analysis

Labeled hearts were analyzed with an Axiozoom V16 macroscope (Carl Zeiss). For the analysis of fluorescent protein expression, a z stack was realized in each channel. The algorithm extended depth of focus of Zen Blue software (Carl Zeiss) was used to produce two-dimensional (2D) images, and the data were then merged. The SA covered by each cluster of a clone was measured using ImageJ software (Schindelin et al., 2012).

Clonal Volume Analysis

For whole-mount confocal microscopy, hearts were cleared with ScaleCUBIC-1 and ScaleCUBIC-2 solutions as described in Susaki et al. (2014). Hearts were incubated overnight at room temperature (RT) in ScaleCUBIC-1 solution and incubated at RT in ScaleCUBIC-2 solution for at least 2 days before their analysis. The whole-mount acquisitions were acquired with a biphoton confocal microscope (LSM780; Carl Zeiss). SA and volume occupied by a single *Mesp1*-expressing cells were analyzed with ImageJ software (Schindelin et al., 2012).

SA Analysis of Mosaically Labeled Hearts

Mesp1-Cre mice were crossed with the *Rosa-Confetti* reporter mice. E12.5, P1, and 6-month-old hearts were fixed in 4% paraformaldehyde for 1 to 3 hr, depending on their stage at RT. Counterstaining of nuclei was performed with Topro3 (1/500, Invitrogen). The surface acquisitions were acquired with a confocal microscope (LSM780; Carl Zeiss). SA on maximum intensity projection was measured using Fiji software (Schindelin et al., 2012).

SUPPLEMENTAL INFORMATION

Supplemental Information includes Supplemental Theory, Supplemental Experimental Procedures, and one figure and can be found with this article online at http://dx.doi.org/10.1016/j.celrep.2015.12.013.

AUTHOR CONTRIBUTIONS

All the authors designed the experiments and performed data analysis. S.C. and F.L. performed the mouse experiments and confocal analysis. S.R. and B.D.S. developed the biophysical modeling schemes and performed the statistical analysis. All authors contributed to the writing of the manuscript.

ACKNOWLEDGMENTS

We would like to thank J.-M. Vanderwinden for his help with confocal microscopy. We are grateful to members of the C.B. lab for their comments on the manuscript. S.C. and N.M. are supported by a fellowship from the FRS/FRIA. F.L. has been supported by a EMBO Long-Term Fellowship. B.D.S. and S.R. are supported by Wellcome Trust (grant number 098357/Z/12/Z). C.B. is an investigator of WELBIO. This work was supported by the FNRS, the ULB Foundation, the European Research Council (ERC), and the Bettencourt Schueller Foundation (C.B. and F.L.).

Received: July 20, 2015
Revised: October 7, 2015
Accepted: November 23, 2015
Published: December 24, 2015

REFERENCES

Auman, H.J., Coleman, H., Riley, H.E., Olale, F., Tsai, H.J., and Yelon, D. (2007). Functional modulation of cardiac form through regionally confined cell shape changes. PLoS Biol. *5*, e53.

Bailey, N.T.J. (1964). The Elements of Stochastic Processes (New York: Wiley).

Buckingham, M., Meilhac, S., and Zaffran, S. (2005). Building the mammalian heart from two sources of myocardial cells. Nat. Rev. Genet. *6*, 826–835.

de Boer, B.A., van den Berg, G., de Boer, P.A., Moorman, A.F., and Ruijter, J.M. (2012). Growth of the developing mouse heart: an interactive qualitative and quantitative 3D atlas. Dev. Biol. *368*, 203–213.

Devine, W.P., Wythe, J.D., George, M., Koshiba-Takeuchi, K., and Bruneau, B.G. (2014). Early patterning and specification of cardiac progenitors in gastrulating mesoderm. eLife *3*, 3.

Garry, D.J., and Olson, E.N. (2006). A common progenitor at the heart of development. Cell *127*, 1101–1104.

Gupta, V., and Poss, K.D. (2012). Clonally dominant cardiomyocytes direct heart morphogenesis. Nature *484*, 479–484.

Koushik, S.V., Wang, J., Rogers, R., Moskophidis, D., Lambert, N.A., Creazzo, T.L., and Conway, S.J. (2001). Targeted inactivation of the sodium-calcium exchanger (Ncx1) results in the lack of a heartbeat and abnormal myofibrillar organization. FASEB J. *15*, 1209–1211.

Lescroart, F., Chabab, S., Lin, X., Rulands, S., Paulissen, C., Rodolosse, A., Auer, H., Achouri, Y., Dubois, C., Bondue, A., et al. (2014). Early lineage restriction in temporally distinct populations of Mesp1 progenitors during mammalian heart development. Nat. Cell Biol. *16*, 829–840.

Meilhac, S.M., Esner, M., Kelly, R.G., Nicolas, J.F., and Buckingham, M.E. (2004a). The clonal origin of myocardial cells in different regions of the embryonic mouse heart. Dev. Cell *6*, 685–698.

Meilhac, S.M., Esner, M., Kerszberg, M., Moss, J.E., and Buckingham, M.E. (2004b). Oriented clonal cell growth in the developing mouse myocardium underlies cardiac morphogenesis. J. Cell Biol. *164*, 97–109.

Perl, A.K., Wert, S.E., Nagy, A., Lobe, C.G., and Whitsett, J.A. (2002). Early restriction of peripheral and proximal cell lineages during formation of the lung. Proc. Natl. Acad. Sci. USA *99*, 10482–10487.

Saga, Y., Miyagawa-Tomita, S., Takagi, A., Kitajima, S., Miyazaki, Ji., and Inoue, T. (1999). MesP1 is expressed in the heart precursor cells and required for the formation of a single heart tube. Development *126*, 3437–3447.

Satou, Y., Imai, K.S., and Satoh, N. (2004). The ascidian Mesp gene specifies heart precursor cells. Development *131*, 2533–2541.

Schepers, A.G., Snippert, H.J., Stange, D.E., van den Born, M., van Es, J.H., van de Wetering, M., and Clevers, H. (2012). Lineage tracing reveals Lgr5+ stem cell activity in mouse intestinal adenomas. Science *337*, 730–735.

Schindelin, J., Arganda-Carreras, I., Frise, E., Kaynig, V., Longair, M., Pietzsch, T., Preibisch, S., Rueden, C., Saalfeld, S., Schmid, B., et al. (2012). Fiji: an open-source platform for biological-image analysis. Nat. Methods *9*, 676–682.

Snippert, H.J., van der Flier, L.G., Sato, T., van Es, J.H., van den Born, M., Kroon-Veenboer, C., Barker, N., Klein, A.M., van Rheenen, J., Simons, B.D., and Clevers, H. (2010). Intestinal crypt homeostasis results from neutral competition between symmetrically dividing Lgr5 stem cells. Cell *143*, 134–144.

Susaki, E.A., Tainaka, K., Perrin, D., Kishino, F., Tawara, T., Watanabe, T.M., Yokoyama, C., Onoe, H., Eguchi, M., Yamaguchi, S., et al. (2014). Whole-brain imaging with single-cell resolution using chemical cocktails and computational analysis. Cell *157*, 726–739.

Tam, P.P., and Beddington, R.S. (1992). Establishment and organization of germ layers in the gastrulating mouse embryo. Ciba Found. Symp. *165*, 27–41, discussion 42–49.

Underwood, E.E. (1970). Quantitative Stereology (Addison-Wesley).

Wessels, A., and Sedmera, D. (2003). Developmental anatomy of the heart: a tale of mice and man. Physiol. Genomics *15*, 165–176.

Wicksell, S.D. (1925). The corpuscle problem. A mathematical study of biometric problem. Biometrika *17*, 84–99.

Cell Reports
Supplemental Information

Uncovering the Number and Clonal Dynamics of *Mesp1* Progenitors during Heart Morphogenesis

Samira Chabab, Fabienne Lescroart, Steffen Rulands, Navrita Mathiah, Benjamin D. Simons, and Cédric Blanpain

Chabab et al. Supplemental Figure S1

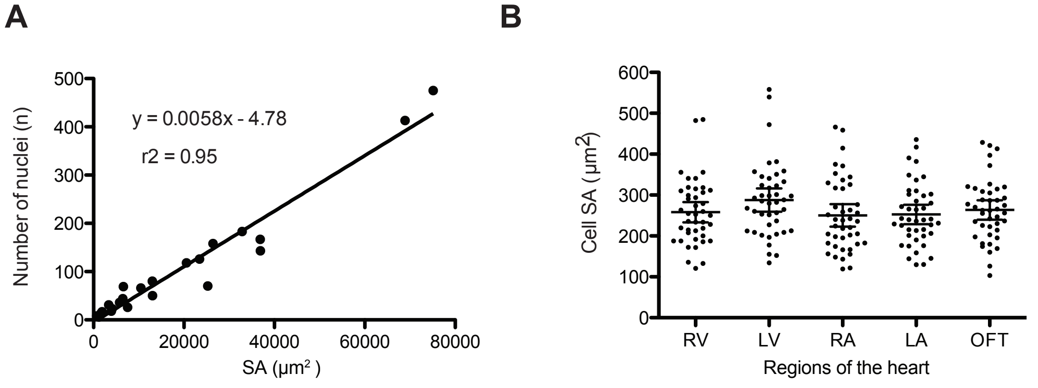

Supplemental Figure S1: Correlation between the surface area and the number of nuclei, related to Figure 1, 2 and 4

A. Correlation between the surface area (SA) (in µm^2) covered by a patch in E12.5 *Mesp1-Cre/Rosa-Confetti* hearts and the number of nuclei (n). The correlation is linear. **B**. Distribution of the measurments of the SA of CFP-expressed cells (+/- 95% CI) (in µm^2) equally distributed along the depth of the heart in the different regions of the *Mesp1-Cre/Rosa-Confetti* hearts at E12.5 showing the size of cardiac cells is constant in the different cardiac regions.

Chabab et al. Supplemental Theory: Analysis of clonal surface areas, related to Figure 1, 2 and 4

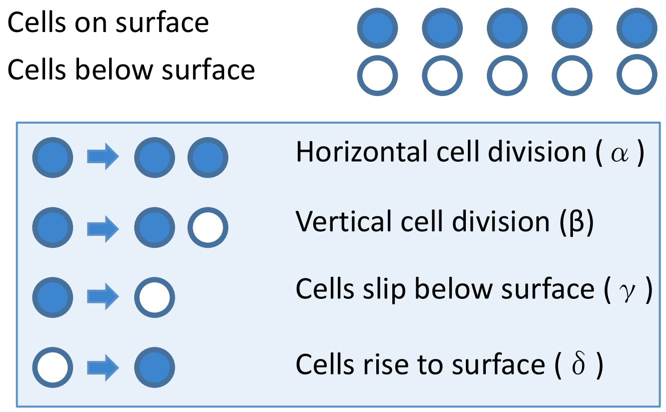

Inferring progenitor fate behaviour from surface-area data, related to Figure 1 and 4

The analysis of clonal dynamics in developing tissues typically relies on the acquisition of a statistical ensemble of clonal data. However, in three-dimensional tissues, the recovery of clonal information requires the reconstruction of serial sections, which is challenging, slow and potentially unreliable. By contrast, the distribution of clone sizes as measured by their "footprint" on the surface of tissues can be acquired at high definition from confocal microscopy of thin sections. Unfortunately, the reconstruction of total clone sizes from random sections through three-dimensional clones requires making detailed assumptions about their morphology, a canonical and unsolvable problem known in the literature as the *corpuscle problem.* Nevertheless, by focusing on cell dynamics at the surface, in this study we show that information on developmental processes can still be recovered.

To this end, we propose a simple and generic modelling framework that allows qualitative insights to be drawn from surface area data. In particular, for an equipotent population of developmental precursors, there are only a limited number of processes that change the number of cells in a labelled clone at the surface: The number of labelled progenitor cells, S, in the surface layer can increase due to tangential (in-plane) cell division, or by labelled cells

being transferred from the bulk into the surface layer (see schematic and Figure 4A). We denote the combined rate of these processes as α. Further, labelled cells may divide perpendicular to the surface so that one daughter cell stays in the surface while the other ends up below the surface (at a rate β). Finally, labelled cells may slip below the surface without division (at a rate γ), while new surface labelling might be initiated continuously as cells labelled in the bulk reach the surface at a rate δ. We can summarize this simple model in chemical notation as the following stochastic process

$$S \xrightarrow{\alpha} SS, \qquad S \xrightarrow{\beta} S\emptyset, \qquad S \xrightarrow{\gamma} \emptyset, \qquad \emptyset \xrightarrow{\delta} S$$

where $\emptyset$ denotes a "vacancy". In the parlance of population dynamics, the behaviour of labelled cell clusters in the surface layer is then effectively described by a "Galton-Watson process with immigration" {Bailey, 1964}. If the timing between consecutive events is random, and statistically uncorrelated (Markovian) with the defined average rate, and fate decisions are cell-autonomous, the chance, $P(n,t)$, of finding a labelled cell cluster with n cells is governed by a *Master equation* of the form,

$$\frac{d}{dt}P(n,t) = [\alpha\,(n-1)+\delta]P(n-1,t) + \gamma(n+1)P(n+1,t)$$
$$-[(\alpha+\gamma)n+\delta]P(n,t).$$

Solving this equation, one finds that $P(n,t)$ is defined by a negative binomial distribution,

$$P(n,t) = \binom{\delta+n-1}{n}(1-b_t)^{\delta} b_t^n,$$

where the time-dependent parameter

$$b_t = \frac{\exp[(\alpha - \gamma)t] - \alpha}{\exp\,[(\alpha - \gamma)t] - \beta}.$$

By contrast, if the timing of division of sister cells were highly synchronized, the surface area distribution would be dominated by the immigration process, leading to a distribution of the form 1/n. However, as is discussed below, in typical clonal datasets the details of cell fate decisions might be hidden in fluctuations stemming from migration processes between cell layers.

As the processes in this model do not translate to precisely defined biological processes, the inferred rates cannot easily be related to the underlying biological parameters such as the true cell division or cell migration (diffusion) rates. Rather, our approach allows the hypothesis of progenitor equipotency to be assessed, and different modes of division to be distinguished on the basis of surface clonal data alone. Note, however, that within the variability of typical clonal datasets, we cannot rule out a potential additional contribution from cell divisions leading to asymmetric fate outcome, which would simply lead to a rescaling of model parameters.

Fitting of the model, related to Figure 4

To fit the model we estimated the number of cells in a clone by dividing its surface area by the average surface area covered by cardiomyocytes (269 ± 13 (95% CI) μm^2). We then employed Maximum Likelihood Estimation as implemented in the nbinfit function of Matlab's® Statistics and Machine Learning Toolbox™. The fitted parameter values were $\delta = 4.2, 4.2, 3.8$ and $\beta_t = 0.7, 1, 0.9$ for the time points E6.25, E6.75 and E7.25, respectively.

Estimation of the number of *Mesp1* progenitors from clonal data, related to Figure 1

To estimate the number of *Mesp1* progenitors contributing to heart development we considered the portion of clone touching the surface as a random slice through the full, three-

dimensional clone. Inferring the distribution of full clone sizes from these slices is mathematically impossible, a manifestation of the *corpuscle problem*. However, assuming that the surface area of a clone is statistically representative of any other section, the average surface area fraction is an unbiased estimator of average volume fraction of a clone in the myocardium (e.g. Underwood, 1970). In other words, the average fraction of labeled *Mesp1* progenitors contributing to the surface in a clone equals the fraction of all labeled *Mesp1+* precursors among all cardiac cells. Hence, while we cannot infer the distributions of clone sizes from surface area data, the fraction of the *average* clone size of all cardiomyocytes is equal to the average percentage of surface area, A_s, covered by a single clone. Importantly, we may therefore identify A_s with the size of a single clone. With this definition, the number of *Mesp1* progenitors contributing to the surface is then given by $N_{Mesp1} = 1/A_s$.

To calculate A_s we again make use of the 89 groups of patches, which we identified as monoclonal by statistical inference (Lescroart et al., 2014). In our previous study, hearts with three or less patches in a given color were assigned as monoclonal. However, in the assignment of hearts that are monoclonal we estimated that some 12% of these were falsely assigned as monoclonal, and likely induced in more than a single clone in a given color (Lescroart et al., 2014). To take this into account, we write the average size of visible clones in "monoclonal" hearts, A_m, as $A_m = \sum_{n=1}^{3} n\, r_n\, A_s$, where r_n denotes the fraction of hearts, which were assigned as monoclonal and were induced with *n* cells. Correspondingly, we find

$$A_s = \frac{A_m}{\sum_n n\, r_n}.$$

The frequencies r_n are given by $r_n = \sum_k L(n|k)F(k)$, with the $L(m|k)$ defining the probability of finding *n* induction events given *k* patches, and the overall frequency of hearts with *k* patches, $F(k)$, as derived in (Lescroart et al., 2014). With $A_m = 0.57 \pm 0.05$ % (all errors represent 95% CI), we obtain an average clone size of 0.5% of all CMs. Taken alone this would suggest that about 200 Mesp1+ progenitors are required to cover the total surface of a heart (1/0.005).

We next take into account the fact that some 18% of fragments do not touch the surface of the heart and have therefore not been included in the estimate of the average clone size. Consequently, if the proximity of fragments to the surface is independent of the fragment size, we underestimated the clone size by the same amount. Dividing the average clone size by 82 $\pm$ 9% (95% CI) we therefore obtain a total number of 244 +/- 26 (95% CI) (200/0.82 progenitors) *Mesp1* progenitors.

Calculation of errors in the estimation of progenitors from clonal tracing experiments, related to Figure 1

To calculate the 95% confidence interval for the number of *Mesp1* progenitors we estimated the binomial confidence interval of the proportion of surface touching cells using the Clopper-Pearson method as implemented in Matlab's binofit function (Statistical Toolbox). The relative error associated with the size of monoclonal groups of clusters was less than 2%, such that we could neglect this source of error. We obtained the final estimate for the 95% confidence intervals using the conventional formulas of error propagation (e.g. Clifford, 1973).

Estimation of the number of *Mesp1* progenitors from mosaic tracing experiments, related to Figure 2

We may also infer the number of *Mesp1* progenitors from the number of patches in mosaic labeled hearts. In this case the analysis is complicated by the fact that clones not only fragment but also merge due to a high induction frequency. In previous work, we estimated the fragmentation rate of clones to be $f = 1.6 \pm 0.2$ (95% CI) fragmentation events between labeling and analysis at E12.5, which was roughly independent of the induction time. In other words, a single induction event leads, on average, to 2.6 $\pm$ 0.2 (95% CI) patches.

Merging of clones can occur by different mechanisms: initially distant clones can become neighbors due to tissue remodeling, or cells are labeled in close proximity by chance. Given the high induction frequency we assume that the latter mechanism is by far dominant. To find the rate of labeled mergers, we made use of one of the most studied models in statistical physics. We considered an array of hexagonal cells, which are arranged on a triangular lattice (Figure 2L). Each of these cells is labeled with a probability p and remains unlabeled otherwise. In the resulting pattern, neighboring cells are often by chance labeled in a common color, such that cluster of labeled cells of varying size can emerge. The number of labeled clusters, n, is then given by the number of labeled cells divided by the average cluster size. To estimate the average size of labeled clusters we performed Monte Carlo simulations of the site percolation problem on a hexagonal lattice with 1e6 sites (Stauffer and Aharony, 1992). The average induction probability for hearts harvested at E12.5 was 0.26 ± 0.05 (95% CI), which according to the simulation, corresponds to an average initial cluster size of 2.75 ± 0.05 (95% CI) cells. With this value, the number of *Mesp1* progenitors contributing to heart development is

$$N = \frac{n\, m}{p\, (f + 1)\, s},$$

where n is the number of patches in a given colour and heart, m is the average initial cluster size, f is the fragmentation rate, p is the induction probability (chimerism), and s is the fraction of surface-touching patches among all patches. Finally, we obtain the estimate that 257±24 (95% CI) *Mesp1* progenitors contribute to heart development.

Calculation of errors in the estimation of progenitors from mosaic tracing experiments, related to Figure 2

The calculations of the number of *Mesp1* progenitors and the corresponding uncertainty (95% confidence interval) were performed separately for each colour and each heart. The reported value is the average of these values. The calculations resulting in the induction frequencies

and of the average initial cluster size by Monte Carlo simulations involved large sample sizes, such that we could neglect these sources of error. Since we did not perform the Monte Carlo simulations for each colour and heart separately but for the average induction frequency in all hearts, the uncertainty in the average cluster size was estimated by the uncertainty in this average. Further, the number of patches in each colour and each heart was modelled by a Poisson distribution. The corresponding uncertainty in these estimates was obtained using Matlab's poissfit function from the Statistical Toolbox. The uncertainty of the fragmentation rate was taken from (Lescroart et al., 2014). As the different sources of error are statistically independent the uncertainties for each colour and each heart were propagated using standard formulae. Similarly, standard formulae have been used to calculate uncertainty of the final estimate from the individual values (e.g. Clifford, 1973).

Estimation of cell division rates from the clonal induction to E12.5, related to Figure 4

To determine the overall (all) and horizontal (parallel to surface) proliferation rate of *Mesp1* progenitors we respectively make use of the 8 clones in which the volume was measured and 89 clones in which the surface data was recorded, and that were identified to be monoclonal by statistical inference (Figure 4F). We begin with estimating the overall proliferation rate from the volumes of clones induced at time points E6.75 and E7.25. With the volume of a single cell at the time of analysis roughly given by $v \approx 2150\ \mu m^3$ (de Boer et al., 2012), the number of cells in a clone of volume V is given by $N = V/v$. If all progeny of the precursor labeled at time t_0 divide symmetrically, the size of clones grows exponentially, $N(t) = \exp[\lambda(t - t_0)]$, where λ is the average rate of cell division. With this, we obtain $\lambda = \ln N(t)/(t - t_0)$.

By following a similar approach we can in addition obtain the cell division rate tangential to the heart surface. For this we took the average surface area covered by a single cell to be $s = 269 \pm 13$ (95% CI) μm^2, as was measured with Fiji software (Schindelin et al., 2012) on several optical sections of mosaically labeled hearts (Figure S1B). We calculated the number

of cells within the SA covered by a clone by using the formula N = SA/s. The average SA is determined solely by cell divisions parallel to the surface. Since in a continuously expanding tissue the amount of vertical cell movements in and out of the surface layer must, on average, be equal we could neglect their contribution to the average clonal SA. With this, the "horizontal" proliferation rate of *Mesp1* expressing cells is simply given by the logarithm of the average number of surface-touching cells in a clone, divided by the time span between labeling and analysis. The "horizontal" proliferation rate is around 0.7 times per day and represents some 60-70% of all cell division (Figure 4F).

Supplemental experimental procedures

Estimation of chimerism in mosaically labeled heart

The proportion of fluorescent protein labeled cells expressed at the surface of the heart was analyzed for each channel on maximum intensity projection treated images with Zen Black software (Carl Zeiss, Inc.). The whole surface area (SA) covered by each fluorescent protein relative to the total surface of the heart was measured using Fiji software (Schindelin et al., 2012). Then, the same heart was sectioned (20-µm thick cross-sections) and the same strategy was used to determine the chimerism that occurs inside the heart.

Correlation between the number of nuclei and the surface area of a 2D-labeled cluster

The number of nuclei (counterstained with Topro-3) inside a defined cluster was counted and correlated to the SA of this cluster with Fiji software (Schindelin et al., 2012) on several optical sections of mosaically labeled hearts at E12.5 and P1.

Estimation of cardiac cells number

10 hearts from E13.5 embryos were dissected and digested individually in collagenase (3.5mg/ml) during 1.5 hour at 37°C. Enzyme activity was stopped using the final concentration of 5mM EDTA (Invitrogen). Digestion into single cells was checked microscopically and by FACS analysis where more than 80% of the cell suspension was composed by single cells. The number of cardiac cells was counted on a cell chamber counting (Neubawer, Inc) and validated with a mini automated cell counter (Moxi Z, Orflo technologies).
